# Factors Associated With COVID-19 Non-Vaccination in Switzerland: A Nationwide Study

**DOI:** 10.3389/ijph.2023.1605852

**Published:** 2023-05-22

**Authors:** Serena Sabatini, Marco Kaufmann, Marta Fadda, Stefano Tancredi, Nazihah Noor, Bernadette W. A. Van Der Linden, Stéphane Cullati, Irene Frank, Gisela Michel, Erika Harju, Chantal Luedi, Anja Frei, Tala Ballouz, Dominik Menges, Jan Fehr, Philipp Kohler, Christian R. Kahlert, Victor Scheu, Natalia Ortega, Patricia Chocano-Bedoya, Nicolas Rodondi, Silvia Stringhini, Hélène Baysson, Elsa Lorthe, Maria Caiata Zufferey, L. Suzanne Suggs, Emiliano Albanese, Julia Vincentini, Murielle Bochud, Valérie D’Acremont, Samira Gonseth Nusslé, Medea Imboden, Dirk Keidel, Melissa Witzig, Nicole Probst-Hensch, Viktor von Wyl

**Affiliations:** ^1^ Instutite of Public Health, Università della Svizzera Italiana, Lugano, Switzerland; ^2^ Epidemiology, Biostatistics and Prevention Institute, University of Zurich, Zurich, Switzerland; ^3^ Population Health Laboratory (#PopHealthLab), University of Fribourg, Fribourg, Switzerland; ^4^ Department of Readaptation and Geriatrics, University of Geneva, Geneva, Switzerland; ^5^ Clinical Trial Unit, Lucerne Cantonal Hospital, Lucerne, Switzerland; ^6^ Faculty of Health Sciences and Medicine, University of Lucerne, Lucerne, Switzerland; ^7^ ZHAW Zurich University of Applied Sciences, School of Health Sciences, Winterthur, Switzerland; ^8^ Cantonal Hospital St. Gallen, Division of Infectious Diseases and Hospital Epidemiology, St. Gallen, Switzerland; ^9^ Children’s Hospital of Eastern Switzerland, Division of Infectious Diseases and Hospital Epidemiology, St. Gallen, Switzerland; ^10^ Department of General Internal Medicine, Inselspital, Bern University Hospital, University of Bern, Bern, Switzerland; ^11^ Institute of Primary Health Care (BIHAM), University of Bern, Bern, Switzerland; ^12^ Unit of Population Epidemiology, Division of Primary Care Medicine, Geneva University Hospitals, Geneva, Switzerland; ^13^ Department of Health and Community Medicine, Faculty of Medicine, University of Geneva, Geneva, Switzerland; ^14^ University Center for General Medicine and Public Health, University of Lausanne, Lausanne, Switzerland; ^15^ Department of Business Economics, Health and Social Care, University of Applied Sciences and Arts of Southern Switzerland, Manno, Switzerland; ^16^ Institute of Communication and Public Policy, Università della Svizzera Italiana, Lugano, Switzerland; ^17^ University Centre for Primary Care and Public Health (Unisanté), University of Lausanne, Lausanne, Switzerland; ^18^ Swiss Tropical and Public Health (TPH) Institute, Allschwil, Switzerland; ^19^ Faculty of Medicine, University of Basel, Basel, Switzerland; ^20^ Institute for Implementation Science in Health Care, University of Zurich, Zurich, Switzerland

**Keywords:** COVID-19, SARS-CoV-2, preventive measures, vaccination acceptance, attitudes and beliefs

## Abstract

**Objectives:** We compared socio-demographic characteristics, health-related variables, vaccination-related beliefs and attitudes, vaccination acceptance, and personality traits of individuals who vaccinated against COVID-19 and who did not vaccinate by December 2021.

**Methods:** This cross-sectional study used data of 10,642 adult participants from the Corona Immunitas eCohort, an age-stratified random sample of the population of several cantons in Switzerland. We used multivariable logistic regression models to explore associations of vaccination status with socio-demographic, health, and behavioral factors.

**Results:** Non-vaccinated individuals represented 12.4% of the sample. Compared to vaccinated individuals, non-vaccinated individuals were more likely to be younger, healthier, employed, have lower income, not worried about their health, have previously tested positive for SARS-CoV-2 infection, express lower vaccination acceptance, and/or report higher conscientiousness. Among non-vaccinated individuals, 19.9% and 21.3% had low confidence in the safety and effectiveness of SARS-CoV-2 vaccine, respectively. However, 29.1% and 26.7% of individuals with concerns about vaccine effectiveness and side effects at baseline, respectively vaccinated during the study period.

**Conclusion:** In addition to known socio-demographic and health-related factors, non-vaccination was associated with concerns regarding vaccine safety and effectiveness.

## Introduction

The development and uptake of effective vaccines against severe acute respiratory syndrome virus 2 (SARS-CoV-2) and its related disease COVID-19 led to notable reductions in hospitalizations, and excess mortality [1–3]. In Switzerland, SARS-CoV-2 vaccination campaigns started in December 2020 and initially targeted high-risk populations such as older people, individuals with underlying conditions and disabilities, and healthcare workers. Since May 2021, everyone aged 50+ years could get the vaccine and, since June 2021, the vaccine also became available for those aged 16–49 years [4]. Towards the end of 2021, individuals who had received the first series of vaccines against SARS-CoV-2 could receive a booster vaccination.

By end of 2021 about 20.1% of Swiss adults were not vaccinated against SARS-CoV-2 [5]. As non-vaccinated individuals are at greater risk of experiencing a more severe course of the SARS-CoV-2 infection with advancing age [6], increasing vaccination uptake in this subgroup is of pivot importance [7]. Local, national, and global public health authorities highly recommend vaccination [4, 7]. However, the effectiveness of current strategies promoting vaccination uptake is unclear, and the reasons for the hesitancy of certain eligible individuals are poorly understood. Evidence is needed to inform ongoing campaigns aimed to promote a second SARS-CoV-2 booster of the bivalent vaccine developed to target the rapidly spreading Omicron subvariants [8–11].

Before the SARS-CoV-2 vaccine became available, numerous studies explored individual’s hesitancy towards a potential vaccine worldwide [12–22]. Although most individuals expressed their intention to take up COVID-19 vaccinations upon availability (e.g., 61% in the United States according to [23] and 68% in Switzerland according to [13]) a minority were likely to be hesitant towards vaccination.

The individuals in the hesitant groups were more likely to be younger, women, unemployed, part of an ethnic minority, religious, have low levels of education, and lower income [12–19]. Lower scores on the personality traits of openness, agreeableness, conscientiousness, and/or higher levels of neuroticism, were also associated with greater vaccination hesitancy, but associations were of small magnitude [20–22]. Individuals less afraid of SARS-CoV-2 [24], possibly due to prior infection [13], or those assuming that one’s own immune system would be sufficient to fight the infection [25] reported greater vaccination hesitancy. Vaccine hesitancy was also higher among individuals with low trust towards the healthcare system, low confidence in the vaccine’s effectiveness, and/or afraid of the vaccine’s side effects [13–15, 25, 26]. In previous studies these individuals did not exclude the possibility of getting vaccinated once more evidence on vaccine effectiveness and side-effects became available [13, 27].

As the intention to get vaccinated does not always lead to vaccination uptake [28, 29], it remains unclear which factors are associated with actual vaccine uptake or non-vaccination (i.e., vaccine hesitancy). This study aimed to examine whether and to what extent socio-demographic and personal characteristics (e.g., chronic health conditions), worry for one’s health, report of positive COVID-19 test in the participant, health-related variables, vaccination-related beliefs and attitudes, vaccination acceptance, and personality traits were associated with vaccination status.

## Methods

### Source Population and eCohort Study Recruitment

This study used data of individuals aged 20+ from the Corona Immunitas digital follow-up cohort (eCohort) which is part of the Corona Immunitas seroprevalence study, described elsewhere [30, 31]. The source population for the eCohort consisted of participants from the Corona Immunitas study who were randomly selected from the general Swiss population. At enrollment into the Corona Immunitas seroprevalence study, participants completed an entry assessment covering socio-demographic and health factors. Enrollments into Corona Immunitas occurred between June 2020 and February 2021, depending on site [31]. Enrolled participants aged 20+ were also systematically invited to join the longitudinal eCohort if they had a working email address and internet access. Compared to the Corona Immunitas cohort, participants enrolled in the eCohort were more likely to be older, to have a Swiss citizenship, to have a university diploma, and to have greater household income than the general Swiss Population [31]. Further details on eCohort recruitment, survey response and survey topics can be found elsewhere [31].

### Data Collection

Our analysis used data from 11 study sites representing all language regions in Switzerland (i.e., Bern; Basel-Landschaft; Basel-Stadt; Fribourg; Graubünden; Luzern; Neuchâtel; St. Gallen; Ticino; Vaud; and Zürich). We included information from the Corona Immunitas entry assessment, as well as data from the monthly eCohort follow-up surveys. These follow-up surveys collected information on SARS-CoV-2 infections, mental and physical health, healthcare utilization, questions on perceived consequences of the pandemic, perceptions of personal and societal risks concerning the pandemic; and use of the Swiss digital proximity tracing app (i.e., SwissCOVID) [32]. Starting in February 2021, the monthly surveys also included additional questions about SARS-CoV-2 vaccination status, as well as questions on attitudes and hesitancy towards vaccination, which were only presented to people who had not yet received a vaccine dose. Additional data on personality traits were collected using a separate survey at a single time point between March 2021 and April 2021, depending on eCohort site.

### Selection of Participants for Analysis

**FIGURE 1:**
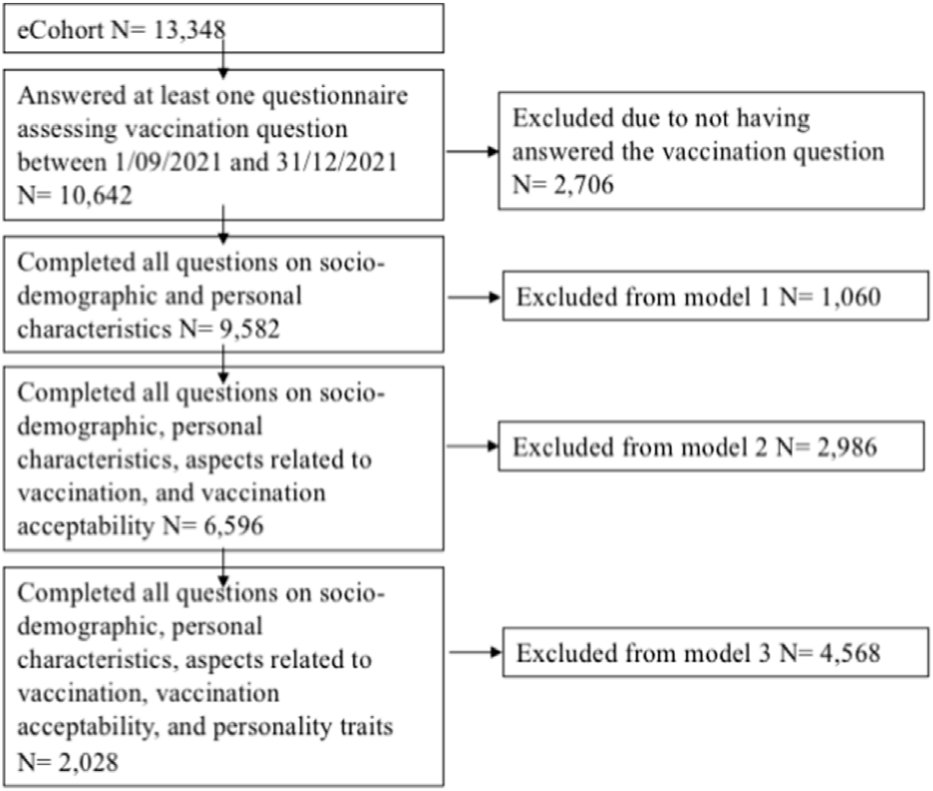
Flow chart of analytic samples. Corona Immunitas eCohort. Switzerland. February–December 2021.

This analysis included participants who filled out at least one vaccine status assessment as part of an eCohort follow-up survey between 1 September 2021 and 31 December 2021 (Figure 1). The analysis baseline was set for each individual at the first available vaccination status survey. Baseline information included age, gender, education, number of health conditions, work status, income, and language region from the entry assessment for Corona Immunitas, which needed to be non-missing for inclusion in analysis. Moreover, attitudes toward vaccination and vaccine hesitance were taken from the monthly survey that defined the individual analysis baseline. The earliest possible baseline date was 1 February 2021 (when the first monthly vaccination survey was released), and the maximally possible analysis follow-up duration was 11 months (i.e., from February to December 2021).

### Study Measures

All characteristics were self-reported.

Vaccination status (1 = Non-vaccinated; 0 = Vaccinated) was self-reported by participants at each monthly follow-up. We created a variable Vaccinated which means having received at least one vaccine dose by the end of 31 December 2021.

### Socio-Demographic and Personal Characteristics

Socio-demographic and personal characteristics were reported in the Corona Immunitas entry assessment. Age was expressed in years. Gender comprised three categories: men; women; other. Education comprised three categories: primary (i.e., No school certificate; Mandatory school); secondary (i.e., Apprenticeship); and tertiary education (i.e., Higher technical school; University of applied sciences; University). Household monthly gross income comprised four categories: Swiss Francs, CHF: 0–6,000; >6,000–12,000; >12,000–18,000; >18,000. Language spoken in the study site was coded as German (Bern; Basel-Landschaft; Basel-Stadt; Graubūnden; Luzern; St. Gallen; Zürich; and 1/3 of the sample in Fribourg); French (2/3 of the sample in Fribourg; Neuchâtel; Vaud); and Italian (Ticino). Work status comprised three categories: 1 = Employed; 2 = Retired; 3 = Not employed and not retired.

### Health-Related Variables

#### Number of Chronic Health Conditions

We coded the count of self-reported diagnoses of cancer; diabetes; immunological disease or treatment with immunosuppressant; hypertension; cardiovascular disease; and other diseases not listed (each counted as one).

#### Worry for One’s Health

At Corona Immunitas entry assessment, individuals indicated how much they worry about the consequences of the current situation on their health (1 = Not at all; 5 = Extreme). This variable was treated as categorical.

#### Prior Positive SARS-CoV-2 Test in the Participant

At each follow-up assessment participants reported whether they had done a SARS-CoV-2 test and, if so, what the result was. Based on retrospective information until 31 December 2021, and prior to participants’ vaccination, we created a dichotomous variable reporting whether, among those who reported having done a SARS-CoV-2 test, participants ever reported a positive SARS-CoV-2 test (0 = No; 1 = Yes).

Vaccination-related beliefs and attitudes were assessed at analysis baseline (defined by the first vaccination status survey) and solely among those who had not yet been vaccinated before baseline assessment. We used 11 questions based on previous studies by Fadda, Albanese [33], Lazarus, Ratzan [34], Neumann-Böhme, Varghese [35], Larson, Schulz [36]. Participants answered to each question on a 5-point Likert scale (1 = Strongly disagree; 5 = Strongly agree).

Vaccination acceptance was assessed at analysis baseline with five questions from the Swiss adaptation of the Vaccination Acceptance Index [13]. Questions were answered on a 5-point Likert scale measuring agreement (1 = Totally disagree; 5 = Totally agree). A total score was calculated by summing up scores on individual items (possible range: 5–25); higher scores indicate greater vaccination acceptance.

#### Personality Traits

The Big Five Inventory was administered once per study site as a separate survey to assess the five classic personality traits of extraversion, openness, conscientiousness, agreeableness, and neuroticism [37]. Each personality trait was assessed with two items. For each item participants indicated their degree of agreement (1 = Disagree strongly; 5 = Agree strongly). One item for each personality trait was reverse-coded in a way that higher scores indicated greater presence of the given personality trait. Higher total scores (possible range: 2–10) indicate a greater presence of the given trait [37].

### Statistical Analysis

For both vaccinated and non-vaccinated participants, we descriptively analyzed socio-demographic and personal characteristics, health-related variables, vaccination-related beliefs and attitudes, vaccination acceptance, and personality traits at baseline. We used univariable and multivariable logistic regression models to estimate the likelihood of remaining non-vaccinated until 31 December 2021 according to analysis baseline socio-demographic and personal characteristics, health-related variables, vaccination acceptance, and personality traits. Based on evidence from other studies, age, gender, education, and number of health conditions were included *a priori* as predictors of vaccination status in the multivariable logistic regression model [12–19]. Each additional variable from demographic and personal characteristic, health-related variables, vaccination acceptance, and personality traits was added to this model sequentially and maintained in the model if the Akaike’s Information Criterion (AIC) decreased by two or more units [38]. As some questions were only answered by a subsample of participants, we calculated three multivariable models for socio-demographic and personal characteristics (model 1/basic model); health-related variables and vaccination acceptance (model 2, building on top of model 1), personality traits (model 3, building on top of model 2). In each model complete case analysis was used. A sensitivity analysis was also conducted using the sample size of model 3 to repeat estimation of models 1 and 2 (see Supplementary Tables S1, S2). Descriptive statistics reporting the socio-demographic characteristics of the overall study sample and of the subsample of participants with full data in all study variables are reported in Supplementary Table S3. Univariable logistic regression models were also fit to report the association of vaccination status with each of the investigated variables. We report odds ratios (OR) and 95% confidence intervals (CIs). Statistical significance was set at a two-tailed value of *p* < 0.05. For categorical variables in the models, the first category was chosen as reference. Data analysis was conducted using Stata version 17 [39].

## Results

### Study Sample

Of the 13,348 eCohort participants, 10,642 (79.7%) had included at least one survey with vaccination questions between 1 September 2021 and 31 December 2021, thereby contributing data to this study. Of these, 1,323 (12.4%) were non-vaccinated by 31 December 2021 (Figure 1).

For our complete case analysis, 9,582 participants were included in Model 1 (basic model) as they had non-missing information on age, gender, education, number of health conditions, work status, income, and language spoken in the study site questions. Model 2 included a subsample of 6,596 individuals who answered all questions assessing worry for one’s health, prior positive SARS-CoV-2 test in the participant, and vaccination acceptance. Model 3 included a more limited subsample of 2,028 individuals who answered all questions assessing personality traits, which was assessed in the eCohort only in March and April 2021 and hence not available for all study participants.

### Baseline Socio-Demographic and Personal Characteristics

Vaccinated participants had, at baseline, a mean age of 57.71 years (Standard deviation, SD = 15.94 years; Table 1) whereas non-vaccinated participants had a mean age of 46.10 years (SD = 15.15 years). Men represented 45.5% of vaccinated participants and 40.4% of non-vaccinated participants. Compared to non-vaccinated individuals, a lower proportion of vaccinated participants completed secondary education (45.7% of vaccinated vs. 52.0% of non-vaccinated), but a higher proportion completed tertiary education (49.5% of vaccinated vs. 43.1% of non-vaccinated). A gross monthly income of CHF 6,000 to 12,000 was reported by a higher proportion of vaccinated individuals (47.1%) compared to non-vaccinated individuals (43.8%). The mean number of chronic health conditions were 0.59 (SD = 0.89) in the vaccinated sample and 0.35 (SD = 0.68) in the non-vaccinated sample. Among vaccinated participants, 30.4% were retired and 43.4% were employed whereas among non-vaccinated participants, 15.1% were retired and 60.6% were employed.

**TABLE 1:** Descriptive statistics for socio-demographic and personal characteristics and estimation of likelihood of being non-vaccinated according to participants’ personal characteristics. Corona Immunitas eCohort. Switzerland. February–December 2021.

	Overall sample (N = 10,642)		Univariable logistic regressions		Multivariable logistic regression^a^ (N = 9,582)	
Variables administered to everyone at baseline	Vaccinated (N = 9,319)	Not vaccinated (N = 1,323)	Odds ratios (95% CI)	*p*-values	Odds ratios (95% CI)	*p*-values
Age, M (SD)	57.71 (15.94)	46.10 (15.15)	0.96 (0.95; 0.96)	<0.001	0.97 (0.96; 0.97)	<0.001
Missing	336	65				
Gender, n (%)						
Women	4,745 (52.7)	732 (57.4)	(Reference)		(Reference)	
Men	4,237 (47.2)	534 (42.5)	0.82 (0.73; 0.92)	0.004	0.99 (0.87; 1.12)	0.726
Other	6 (0.1)	1 (0.1)	1.08 (0.13; 8.99)		1.65 (0.19; 13.98)	
Missing	331	65				
Education, n (%)						
Primary	432 (4.8)	62 (4.9)	(Reference)	0.001	(Reference)	<0.001
Secondary	4,094 (45.2)	651 (52.0)	1.08 0.82; 1.42)		1.06 (0.78; 1.45)	
Tertiary	4,428 (49.5)	540 (43.1)	0.82 (0.62; 1.08)		0.80 (−0.58; 1.10)	
Missing, n	365	70				
Number of chronic health conditions, M (SD)	0.59 (0.89)	0.35 (0.68)	0.65 0.60; 0.71)	<0.001	0.86 (0.78; 0.94)	0.002
Missing, n	936	161				
Work status, n (%)						
Employed	3,903 (43.4)	761 (60.6)	(Reference)	<0.001	(Reference)	<0.001
Retired	3,544 (39.4)	190 (15.1)	0.28 (0.24; 0.33)		0.56 (0.44; 0.71)	
Not employed	1,539 (17.1)	304 (24.2)	1.01 (0.87; 1.17)		1.10 (0.94; 1.29)	
Missing	333	68				
Income, n (%)						
CHF 0–6,000	2,838 (33.7)	499 (42.9)	(Reference)	<0.001	(Reference)	<0.001
CHF >6,000–12,000	3,965 (47.1)	510 (43.8)	0.73 (0.64; 0.83)		0.72 (0.62; 0.83)	
CHF >12,000–18,000	1,123 (13.3)	111 (9.5)	0.56 (0.46; 0.70)		0.47 (0.38; 0.60)	
CHF >18,000	495 (5.9)	44 (3.8)	0.51 (0.37; 0.71)		0.47 (0.33; 0.65)	
Missing, n	900	159				
Language spoken in the study site					n.i.	
German	5,267 (56.5)	784 (59.3)	(Reference)	<0.001		
French	3,238 (34.8)	387 (29.3)	0.80 (0.71; 0.91)			
Italian	814 (8.7)	152 (11.5)	1.25 (1.04; 1.52)			

^a^ Age, gender, education, number of chronic health conditions were covariates included in the models *a priori*. Other predictors were included in the models only when they lead to a decrease in Akaike’s Information Criterion (AIC) of 2 units or more. Odds ratios and *p*-values are reported only for those variables that were kept in the final multivariable model (model 3). For categorical variables we estimated one global *p*-value. n.i.: means not included because has not lead to a decrease in Akaike’s Information Criterion (AIC) of 2 units or more. N: number. CI: Confidence Interval. M: Mean. SD: Standard Deviation. CHF: Swiss Franc.

Amongst all the socio-demographic and personal characteristics variables investigated, in the multivariable logistic regressions (model 1, analytic sample N = 9,582), compared to vaccinated individuals, non-vaccinated individuals were statistically significantly more likely to be younger (OR = 0.97; 95% CI: 0.96; 0.97) and to have fewer chronic health conditions (OR = 0.86; 95% CI: 0.78; 0.94). Non-vaccinated individuals were also less likely to be retired (OR = 0.56; 95% CI: 0.44; 0.71) and less likely to have a higher monthly income (*p* < 0.001). Education did not differ significantly between the two samples.

### Health-Related Variables and Vaccination Acceptance

On average, the non-vaccinated subgroup reported lower levels of vaccination acceptance as indicated by their score on the Vaccination Acceptance Index (mean = 14.09; SD = 4.62; Table 2) than the vaccinated group (mean = 19.93; SD = 3.98).

**TABLE 2:** Descriptive statistics of health-related variables and vaccination acceptance for vaccinated and non-vaccinated individuals and estimation of likelihood of being non-vaccinated according to participants’ answer on health-related variables and vaccination acceptance. Corona Immunitas eCohort. Switzerland. February–December 2021.

	Overall sample (N = 10,642)		Univariable logistic regressions		Multivariable logistic regression (N = 6,596)	
Variables administered to a subsample	Vaccinated (N = 9,319)	Not vaccinated (N = 1,323)	Odds ratios (95% CI)	*p*-values	Odds ratios (95% CI)	*p*-values
Basic model (model 1)^a^						
Worry for one’s health at baseline, n (%)						
Not at all	1,442 (16.1)	430 (34.3)	(Reference)	<0.001	(Reference)	<0.001
A bit	2,738 (30.6)	391 (31.2)	0.49 (0.42; 0.58)		0.66 (0.53; 0.83)	
Moderate	2,969 (33.2)	291 (23.2)	0.34 (0.29; 0.40)	0	0.57 (0.45; 0.72)	
A lot	1,576 (17.6)	118 (9.4)	0.25 (0.20; 0.31)		0.70 (0.51; 0.95)	
Extreme	232 (2.6)	24 (1.9)	0.35 (0.23; 0.56)		0.35 (0.16; 0.75)	
Missing, n	363	69				
Prior positive COVID-19 test in the participant at follow-ups, n yes (%)	321 (3.4)	92 (7.0)	2.08 (1.62; 2.67)	<0.001	2.18 (1.49; 3.19)	<0.001
Vaccination acceptance index at baseline, M (SD)	19.93 (3.98)	14.09 (4.62)	0.76 (0.75; 0.78)	<0.001	0.77 (0.76; 0.79)	<0.001
Missing, n	2,902	447				

^a^ The basic model included age, gender, education, number of health conditions, working status, and income. Other predictors were included in the models only when they lead to a decrease in Akaike’s Information Criterion (AIC) of 2 units or more. For categorical variables we estimated one *p*-value.

Variables were added sequentially to a “basic model”.

N, number; M, Mean; SD, Standard Deviation; CI, Confidence Intervals.

In multivariable logistic regressions (analytic sample N = 6,596; Table 2) adjusted for age, gender, education, number of health conditions, working status, and income, those who were worried for their own health at baseline were more likely to be non-vaccinated than those not at all worried about their own health (*p* < 0.001). Participants who reported having a previous infection determined by a positive SARS-CoV-2 test were more likely to be non-vaccinated than those who did not report a positive test (OR = 2.18; 95% CI: 1.49; 3.19). Non-vaccinated individuals were also more likely to obtain a lower score on the vaccination acceptance index (OR = 0.77; 95% CI: 0.76; 0.79). Based on the final model, we post-hoc also explored interactions of the vaccination acceptance index with age and sex to examine influences of differences in general attitudes towards vaccinations in different subpopulations. While there was no evidence for an interaction with sex (*p* > .05), we observed an interaction between vaccination with increasing age (OR = .98; 95% CI: .97; .99; *p* = .01), suggesting that those who were older were less likely to be non-vaccinated when they had more positive attitudes towards vaccination (higher score on the vaccination acceptability index).

### Personality Traits

The multivariable logistic regression model (model 3, N = 2,028; Table 3) shows that higher levels of conscientiousness were related to greater likelihood of being non-vaccinated (OR = 1.14; 95% CI: 1.01; 1.30).

**TABLE 3:** Descriptive statistics of personality traits for vaccinated and non-vaccinated individuals and estimation of likelihood of being nonvaccinated according to participants’ personality traits. Corona Immunitas eCohort. Switzerland. February–December 2021.

	Sample (N = 3,909)		Univariable logistic regressions		Multivariable logistic regression (N = 2,028)	
Variables administered to a subsample at follow-ups	Vaccinated (N = 3,474)	Not vaccinated (N = 435)	Odds ratios (95% CI)	*p*-values	Odds ratios (95% CI)	*p*-values
Starting model (model 2)^a^						
Extraversion, M (SD)	7.11 (1.70)	7.38 (1.76)	1.10 (1.03; 1.66)	0.002	n.i.	
Missing, n	49	6				
Openness, M (SD)	6.87 (1.68)	6.97 (1.75)	1.03 (0.97; 1.10)	0.262	n.i.	
Missing, n	37	3				
Conscientiousness, M (SD)	8.16 (1.41)	8.87 (1.35)	1.05 (0.98; 1.13)	0.180	1.14 (1.01; 1.30)	0.038
Missing, n	38	7				
Agreeableness, M (SD)	7.74 (1.24)	7.60 (1.32)	0.92 (0.85; 0.99)	0.033	n.i.	
Missing, n	45	7				
Neuroticism, M (SD)	4.84 (1.66)	4.91 (1.73)	1.02 (0.06; 1.09)	0.452	n.i.	
Missing, n	31	4				

^a^ Variables were added sequentially to the starting model including age, gender, education, number of health conditions, working status, income, worry for one’s health, prior positive COVID-19 test in the participant, and scores on the Vaccination Acceptance Index. Other predictors were included in the models only when they lead to a decrease in Akaike’s Information Criterion (AIC) of 2 units or more; n.i. means not included. N= Number. M: Mean. SD: Standard Deviation. CI: Confidence Intervals.

### Vaccination-Related Beliefs and Attitudes at Baseline

Table 4 reports descriptive statistics for vaccination-related beliefs and attitudes at baseline based on the overall study sample (N = 10,642). More than double of non-vaccinated individuals (65.5%) reported (i.e., answered either strongly agreed or agreed) preferring to wait before being vaccinated until more is known about the vaccine’s effectiveness compared to vaccinated individuals (29.1%). Similarly, more than double of non-vaccinated individuals (67.8%) reported preferring to wait before being vaccinated until more is known about the vaccine’s safety compared to vaccinated individuals (31.8%). About half (51.7%) of non-vaccinated individuals feared vaccination side-effects compared to one-fourth of vaccinated individuals (26.7%).

**TABLE 4:** Descriptive statistics of items capturing vaccination-related beliefs and attitudes and vaccination acceptance at baseline for the vaccinated and not vaccinated subsamples. Corona Immunitas eCohort. Switzerland. February–December 2021.

Vaccination-related beliefs and attitudes at baseline			
	Overall sample (N = 10,642)		
Items and answer options	Vaccinated (N = 9,319)	Not-vaccinated (N = 1,323)	*p*-value
	n (%)	n (%)	
I prefer to wait before being vaccinated until more is known about the vaccine’s effectiveness			
Strongly disagree	1,417 (34.6)	139 (11.2)	<0.001
Disagree	756 (18.5)	126 (10.1)	
Neither agree nor disagree	689 (16.8)	165 (13.3)	
Agree	687 (16.8)	246 (19.8)	
Strongly agree	544 (12.3)	569 (45.7)	
^a^Missing, n	5,226	78	
I prefer to wait before being vaccinated until more is known about the vaccine’s safety			
Strongly disagree	1,332 (32.6)	133 (10.7)	<0.001
Disagree	782 (19.1)	114 (9.2)	
Neither agree nor disagree	673 (16.5)	152 (12.3)	
Agree	699 (17.1)	235 (19.0)	
Strongly agree	602 (14.7)	605 (48.8)	
^a^Missing, n	5,231	84	
I believe that vaccination protects me from an infection with the coronavirus			
Strongly disagree	162 (3.9)	227 (18.3)	<0.001
Disagree	309 (7.5)	254 (20.4)	
Neither agree nor disagree	819 (19.9)	366 (29.4)	
Agree	1715 (41.8)	296 (23.8)	
Strongly agree	1,102 (26.8)	100 (8.1)	
^a^Missing, n	5,212	80	
I believe that the vaccination protects me against a severe course of coronavirus infection			
Strongly disagree	102 (2.5)	146 (11.8)	<0.001
Disagree	233 (5.7)	221 (17.8)	
Neither agree nor disagree	614 (15.0)	355 (28.6)	
Agree	1,603 (39.1)	345 (27.8)	
Strongly agree	1,546 (37.7)	175 (14.1)	
^a^Missing, n	5,221	81	
I believe that the vaccination protects against transmission of the coronavirus to others			
Strongly disagree	422 (10.3)	295 (23.8)	<0.001
Disagree	584 (14.3)	192 (23.6)	
Neither agree nor disagree	1,091 (26.7)	351 (28.4)	
Agree	1,276 (31.3)	205 (16.6)	
Strongly agree	710 (17.4)	95 (7.7)	
^a^Missing, n	5,236	85	
I am afraid of possible side effects			
Strongly disagree	1,186 (29.1)	231 (18.6)	<0.001
Disagree	1,005 (24.6)	162 (13.1)	
Neither agree nor disagree	800 (19.6)	206 (16.6)	
Agree	647 (15.9)	244 (19.7)	
Strongly agree	442 (10.8)	396 (32.0)	
^a^Missing, n	5,239	84	
I prefer natural immunity against the coronavirus to vaccine-induced immunity			
Strongly disagree	2,109 (51.8)	185 (15.0)	<0.001
Disagree	600 (14.7)	136 (11.0)	
Neither agree nor disagree	687 (16.9)	231 (18.7)	
Agree	364 (8.9)	217 (17.6)	
Strongly agree	314 (7.7)	466 (37.7)	
^a^Missing, n	5,245	88	
I would rather protect myself by other means (physical distancing, hand hygiene, wearing a mask) than be vaccinated			
Strongly disagree	1891 (46.4)	210 (16.9)	<0.001
Disagree	804 (19.7)	198 (15.9)	
Neither agree nor disagree	798 (19.6)	343 (27.6)	
Agree	350 (8.6)	235 (18.9)	
Strongly agree	230 (5.7)	256 (20.6)	
^a^Missing, n	5,246	81	
Medical reasons (e.g., allergies) prevent me from being vaccinated			
Strongly disagree	3,568 (88.1)	937 (76.1)	<0.001
Disagree	222 (5.5)	104 (8.4)	
Neither agree nor disagree	122 (3.0)	83 (6.7)	
Agree	58 (1.4)	41 (3.3)	
Strongly agree	79 (2.0)	67 (5.4)	
^a^Missing, n	5,270	91	
The coronavirus vaccine has been developed too quickly			
Strongly disagree	1,135 (27.8)	116 (9.4)	<0.001
Disagree	840 (20.6)	98 (7.9)	
Neither agree nor disagree	1,020 (25.0)	253 (20.4)	
Agree	611 (15.0)	270 (21.8)	
Strongly agree	476 (11.7)	502 (40.5)	
^a^Missing, n	5,237	84	
I feel overwhelmed by information on the coronavirus vaccine			
Strongly disagree	1,401 (34.2)	284 (23.0)	<0.001
Disagree	841 (20.6)	214 (17.3)	
Neither agree nor disagree	921 (22.5)	276 (22.4)	
Agree	575 (14.1)	231 (18.7)	
Strongly agree	355 (8.7)	230 (18.6)	
^a^Missing, n	5,226	88	

^a^ Participants with missing data were not asked the questions assessing vaccination-related beliefs and attitudes and vaccination acceptance at baseline as they were already vaccinated at baseline. *p*-values were calculated using chi-squared tests. N: Number.

Non-vaccinated individuals were more likely to distrust public health authorities and vaccination in general. For example, 29.7% of non-vaccinated individuals compared to 68.4% of vaccinated individuals trusted manufacturers or pharmaceutical companies. Moreover, whereas 42.5% of non-vaccinated individuals trusted the Federal Office of Public Health, this institution was trusted by 76.5% of vaccinated individuals.

Among non-vaccinated participants, 31.9% thought that SARS-CoV-2 vaccination protects them from SARS-CoV-2 infection and 41.9% thought that vaccination protects them against a severe course of disease. These proportions amounted to 68.6% and 76.8%, respectively, in vaccinated individuals. Moreover, whereas among non-vaccinated participants, 24.3% thought that vaccination protects them against transmission of the SARS-CoV-2 infection to others, 48.7% of vaccinated individuals thought so. Finally, 55.3% of non-vaccinated individuals preferred natural immunity (i.e., infection-induced immunity) against the SARS-CoV-2 infection over vaccine-induced immunity and 39.5% preferred protecting themselves by other means (e.g., wearing a mask and hand hygiene); these proportions amounted to 16.6% and 14.3%, respectively, in the vaccinated group.

In Supplementary Tables S1, S2 we report sensitivity analyses where all study analyses were conducted on the subsample with full data in all variables of interest. Results of sensitivity analyses were consistent with main study analyses except for the association between the number of chronic health conditions and vaccination status which was no longer statistically significant in the sensitivity analyses. As outlined in Supplementary Table S2, the subsample with full data had, on average, the same number of health conditions as the overall study sample. However, the subsample with full data included a higher proportion of individuals who were employed and who had higher household income.

## Discussion

This study examined whether socio-demographic and personal characteristics, health-related variables, vaccination-related beliefs and attitudes, vaccination acceptance, and personality traits are associated with non-vaccination. In a sample of 10,642 participants, 12.4% remained unvaccinated by end of December 2021. Individuals more likely to remain unvaccinated were younger, had fewer chronic health conditions, were employed, had lower monthly income, were less worried about their own health, were more likely to have reported a positive SARS-CoV-2 test, expressed lower vaccination acceptance, and/or scored higher on the conscientiousness personality trait.

The findings that non-vaccinated individuals were younger, had a lower income, had fewer chronic health conditions, and were less likely to be retired are aligned with previous evidence on vaccine hesitancy [13–15, 26, 40, 41]. In contrast to previous research, vaccination status did not differ between women and men after accounting for several socio-demographic variables, which may suggest that previous observations may be, at least in part, due to lack of control of confounding variables [17, 40]. Also, our observed age- and health status-related vaccination differences may have been influenced by vaccine eligibility and access prioritization. The findings that younger individuals and individuals with fewer chronic health conditions were more likely to be non-vaccinated may be due to vaccination campaigns having started earlier for older individuals, as well as for individuals with chronic health conditions. Nonetheless, by the end of December 2021, all those who wanted to get vaccinated likely had time and opportunity to do so.

In line with existing evidence, scores on personality traits were either marginally statistically significant or not different between vaccinated and non-vaccinated individuals [21, 22, 42] and are thus unlikely to be good predictors for vaccination behavior. By contrast, we observed statistically significant associations between less health worry and greater likelihood of non-vaccination and this association was not age-confounded [24]. Both in our study and previous studies [43], non-vaccinated individuals who reported a previous infection of SARS-CoV-2 were less afraid of being re-infected, which in turn is related to greater likelihood of non-vaccination [44]. Alternatively, this finding may be due to individuals previously infected with SARS-CoV-2 being instructed by Public Health Authorities to wait up to 3 months after infection to get their first vaccine dose. Further research understanding why individuals previously infected with COVID-19 are less willing, and are less likely, to vaccinate is nonetheless warranted.

In Switzerland, the vast majority of administered SARS-CoV-2 vaccines were mRNA-based. These mRNA vaccines were developed and approved very rapidly in comparison to previous vaccines and vaccine delivery technologies [45]. Concerns about novelty and development speed of vaccines were widespread in our study population, which may have contributed to the observed low confidence in the vaccine’s safety, but also its efficacy against infection, transmission, and a severe course of the SARS-CoV-2 infection. The majority of non-vaccinated individuals expressed information needs about the vaccine’s effectiveness, safety, and side-effects, but many respondents were also suggesting that they may change their attitude about vaccination once more information about effectiveness and safety is conveyed. Indeed, in the group of persons who eventually got vaccinated, around 3 of 10 (29.1%) expressed doubts about vaccine effectiveness at baseline. Similarly around 1 in 4 (26.7%) vaccinated individuals had earlier voiced concerns about vaccine side effects. This finding suggests that such concerns can be overcome and there is an opportunity for behavioral changes through communication of efficacy and safety of the SARS-CoV-2 vaccines.

In our study, many non-vaccinated individuals expressed general mistrust towards authorities, including the Swiss Federal Office of Public Health. This hesitation may be ascribed variably to low health literacy, ineffective communication of health authorities, ideological stances [46], social networks, or past experiences with vaccines [40, 47]. Erroneous beliefs towards the safety and effectiveness of SARS-CoV-2 vaccination are potentially modifiable and should be targeted in campaigns promoting COVID-19 primary vaccines and boosters, as well as through discussions about vaccination with trusted healthcare professionals [48]. Numerous studies have shown the safety and effectiveness of SARS-CoV-2 vaccines against severe disease course [49]. Making this evidence more accessible to the general population through the use of simple/lay terminology and science outreach may be one of the many actions helping to increase vaccination uptake [48].

### Strengths and Limitations

This study has several limitations. First, the proportion of non-vaccinated individuals in this study by end of December 2021 (12.4%) is lower than that reported in Switzerland in January 2023 (about 20%) [4] and therefore overrepresented the vaccination rate of the population. It may be that due to social desirability bias, non-vaccinated individuals were more likely to withdraw from the study. However, representativeness of the sample to the overall population was not a requirement for the current study as its main aim was to identify individual-level factors related to non-vaccination. Moreover, the number of non-vaccinated individuals was high enough to ensure sufficient analytical power in all the tested models. Second, direct comparisons between our models cannot be drawn because of the varying analytic samples due to missing values for some exposure variables. However, in the Supplementary Material we report sensitivity analyses where all study analyses were conducted on the subsample with full data in all variables of interest. Although the subsample with full data included a slightly higher proportion of participants who were employed and with better income compared to the overall study sample, sensitivity analyses did not materially alter our conclusions. Only the association between the number of chronic health conditions and vaccination status was no longer statistically significant in the sensitivity analyses. Third, the baseline for participants was set at different timepoints (depending on the completion of the first vaccination survey), and information on efficacy and safety of vaccines may have changed with time. Fourth, vaccination was self-reported by participants and no confirmation was obtained from health authorities.

Nonetheless, this study has several strengths. First, it included extensive assessments of factors related to non-vaccination. Second, the analyses are based on a large sample of individuals aged 20+ years from a representatively selected source population, although subsequent selection effects (e.g., due to selective acceptance of eCohort study enrollment or follow-up participation) cannot be fully excluded. Whereas several research studies focused on the exploration of factors related to willingness to vaccinate against COVID-19 prior to the availability of the vaccine, this study collected data in a period when the SARS-CoV-2 vaccine was available to all adult age groups in Switzerland and the vaccination campaign was ongoing.

### Conclusion

In Switzerland, individuals who are younger, healthier, have a lower income, and/or were previously infected with SARS-CoV-2 were more likely to be non-vaccinated. Individuals who decided not to vaccinate generally had lower confidence in the vaccine’s effectiveness against infection, transmission, and a severe course of the SARS-CoV-2 infection, as well as in the Federal Office of Public Health, and vaccines in general. These findings could inform public health authorities and healthcare providers to target/tailor future vaccination campaigns.
