# Supplementary material for: Factors Associated With COVID-19 Non-Vaccination in Switzerland: A Nationwide Study
Source: Int J Public Health. 2023 May 22;68:1605852. doi: 10.3389/ijph.2023.1605852 (PMC10239801; doi:10.3389/ijph.2023.1605852)
Supplement: Supplementary file 1 [file DataSheet1.docx]

**Supplementary Table 1. Descriptive statistics of socio-demographic, personal characteristics, health-related variables and vaccination acceptance for vaccinated and non-vaccinated individuals and estimation of likelihood of being non-vaccinated according to participants’ personal characteristics and answer on health-related variables and vaccination acceptance.**

|  | Overall sample (N= 2,028) | | Univariable logistic regressions | | Multivariable logistic regression (N= 2,028) | |
| --- | --- | --- | --- | --- | --- | --- |
| **Variables** | Vaccinated  (N= 1,797) | Not vaccinated (N= 231) | Odds ratios  (95% CI) | *p*-values | Odds ratios  (95% CI) | *p*-values |
| Age, M (SD) | 56.34 (15.43) | 44.67 (14.89) | 0.95 (0.95; 0.96) | <0.001 | 0.95 (0.94; 0.96) | <0.001 |
| Gender, n (%) |  |  |  |  |  |  |
| Women | 907 (50.5) | 106 (45.9) | (Reference) |  | (Reference) |  |
| Men | 889 (49.5) | 124 (53.7) | 0.84 (0.64; 1.10) | 0.158 | 1.25 (0.90; 1.73) | 0.295 |
| Other | 1 (0.1) | 1 (0.4) | 7.17 (0.45; 115.35) |  | 13.99 (0.02; 10717.3) |  |
| Education, n (%) |  |  |  |  |  |  |
| Primary | 52 (3.0) | 10 (4.3) | (Reference) | 0.2004 | (Reference) | 0.734 |
| Secondary | 863 (48.0) | 120 (52.0) | 0.72 (0.36; 1.46) |  | 0.72 (0.31; 1.67) |  |
| Tertiary | 882 (49.1) | 101 (43.7) | 0.60 (0.29; 1.21) |  | 0.76 (0.32; 1.82) |  |
| Number of health conditions, M (SD) | 0.58 (0.87) | 0.40 (0.70) | 0.74 (0.61; 0.90) | 0.002 | 1.25 (0.99; 1.57) | 0.064 |
| Missing, n | 0 | 0 |  |  |  |  |
| Work status, n (%) |  |  |  |  | n.i. |  |
| Employed | 858 (47.8) | 159 (68.8) | (Reference) | <0.001 |  |  |
| Retired | 714 (39.7) | 34 (14.7) | 0.26 (0.18; 0.38) |  |  |  |
| Not employed | 225 (12.5) | 38 (16.5) | 0.91 (0.62; 1.34) |  |  |  |
| Income, n (%) |  |  |  |  |  |  |
| CHF 0 – 6'000 | 1456 (81.0) | 199 (86.2) | (Reference) | 0.0001 | (Reference) | 0.019 |
| CHF > 6'000 – 12’000 | 341 (19.0) | 32 (13.9) | 0.54 (0.40; 0.73) |  | 0.59 (0.41; 0.85) |  |
| CHF > 12’000 – 18’000 | 0 | 0 | 0.44 (0.27; 0.72) |  | 0.52 (0.29; 0.92) |  |
| CHF > 18’000 | 0 | 0 | 0.64 (0.33; 1.24) |  | 0.85 (0.39; 1.84) |  |
| Study site |  |  |  |  |  |  |
| German | 1070 (59.5) | 167 (72.3) | (Reference) | 0.0002 | (Reference) |  |
| French | 727 (40.5) | 64 (27.7) | 0.56 (0.42; 0.76) |  | 0.74 (0.52; 1.06) | 0.095 |
| Italian | 0 (0) | 0 (0) |  |  |  |  |
| Worry for one’s health, n (%) |  |  |  |  |  |  |
| Not at all | 206 (11.5) | 75 (32.5) | (Reference) | <0.001 | (Reference) | <0.001 |
| A bit | 550 (30.6) | 74 (32.0) | 0.37 (0.26; 0.53) |  | 0.45 (0.29; 0.68) |  |
| Moderate | 638 (35.5) | 61 (26.4) | 0.26 (0.18; 0.38) |  | 0.41 (0.26; 0.63) |  |
| A lot | 369 (20.5) | 18 (7.8) | 0.13 (0.08; 0.23) |  | 0.28 (0.15; 0.53) |  |
| Extreme | 34 (1.9) | 3 (1.3) | 0.24 (0.07; 0.81) |  | 0.39 (0.10; 1.51) |  |
| Prior positive COVID-19 test in the participant, n yes (%) | 87 (4.8) | 20 (8.7) | 1.86 (1.12; 3.09) | 0.016 | 1.92 (1.05; 3.49) | 0.033 |
| Vaccination acceptance index, M (SD) | 19.57 (3.82) | 14.86 (4.37) | 0.77 (0.75; 0.80) | <0.001 | 0.78 (0.75; 0.81) | <0.001 |
| Conscientiousness, M (SD) | 8.15 (1.31) | 8.30 (1.35) | 1.09 (.98; 1.21) | 0.107 | 1.15 (1.01; 1.31) | 0.031 |

All variables were assessed at baseline except for prior positive COVID-19 test in the participant, vaccination acceptance index, and conscientiousness. Other predictors were included in the models only when they lead to a decrease in Akaike’s Information Criterion (AIC) of 2 units or more; n.i. means not included due to lack of a decrease in Akaike’s Information Criterion (AIC) of 2 units or more.

**Supplementary Table 2. Descriptive statistics of items capturing vaccination-related beliefs and attitudes and vaccination acceptance at baseline for the vaccinated and not vaccinated subsamples for the reduced sample of 2,028 participants**

| **Vaccination-related beliefs and attitudes at baseline** | | | |
| --- | --- | --- | --- |
|  | Overall sample (N= 2,028) | | |
| Items and answer options | Vaccinated  (N= 1,797) | Not vaccinated (N= 231) | p-value |
|  | n (%) | n (%) |  |
| I prefer to wait before being vaccinated until more is known about the vaccine's effectiveness |  |  |  |
| Strongly disagree | 349 (34.3) | 27 (11.8) | <0.001 |
| Disagree | 198 (19.5) | 30 (13.1) |  |
| Neither agree nor disagree | 184 (18.1) | 35 (15.3) |  |
| Agree | 160 (15.7) | 49 (21.4) |  |
| Strongly agree | 127 (12.5) | 88 (38.4) |  |
| ^a^Missing, n | 779 | 2 |  |
| I prefer to wait before being vaccinated until more is known about the vaccine’s safety |  |  |  |
| Strongly disagree | 334 (32.9) | 24 (10.5) | <0.001 |
| Disagree | 207 (20.4) | 29 (12.7) |  |
| Neither agree nor disagree | 176 (17.3) | 32 (14.0) |  |
| Agree | 160 (15.8) | 49 (21.4) |  |
| Strongly agree | 138 (13.6) | 95 (41.5) |  |
| ^a^Missing, n | 782 | 2 |  |
| I believe that vaccination protects me from an infection with the coronavirus |  |  |  |
| Strongly disagree | 45 (4.4) | 37 (16.1) | <0.001 |
| Disagree | 88 (8.6) | 39 (17.0) |  |
| Neither agree nor disagree | 224 (22.0) | 75 (32.6) |  |
| Agree | 463 (45.5) | 64 (27.8) |  |
| Strongly agree | 198 (19.5) | 15 (6.5) |  |
| ^a^Missing, n | 779 | 1 |  |
| I believe that the vaccination protects me against a severe course of coronavirus infection |  |  |  |
| Strongly disagree | 14 (1.4) | 22 (9.6) | <0.001 |
| Disagree | 59 (5.8) | 37 (16.1) |  |
| Neither agree nor disagree | 155 (15.2) | 71 (30.9) |  |
| Agree | 468 (45.9) | 71 (30.9) |  |
| Strongly agree | 323 (31.7) | 29 (12.6) |  |
| ^a^Missing, n | 778 | 1 |  |
| I believe that the vaccination protects against transmission of the coronavirus to others |  |  |  |
| Strongly disagree | 65 (6.4) | 45 (19.7) | <0.001 |
| Disagree | 149 (14.7) | 53 (23.3) |  |
| Neither agree nor disagree | 303 (29.8) | 76 (33.3) |  |
| Agree | 343 (33.7) | 42 (18.4) |  |
| Strongly agree | 157 (15.4) | 12 (5.3) |  |
| ^a^Missing, n | 780 | 3 |  |
| I am afraid of possible side effects |  |  |  |
| Strongly disagree | 229 (22.5) | 14 (6.1) | <0.001 |
| Disagree | 313 (30.7) | 49 (21.4) |  |
| Neither agree nor disagree | 221 (21.7) | 42 (18.3) |  |
| Agree | 160 (15.7) | 60 (26.2) |  |
| Strongly agree | 96 (9.4) | 64 (28.0) |  |
| ^a^Missing, n | 778 | 2 |  |
| I prefer natural immunity against the coronavirus to vaccine-induced immunity |  |  |  |
| Strongly disagree | 485 (47.8) | 32 (14.0) | <0.001 |
| Disagree | 164 (16.2) | 29 (12.7) |  |
| Neither agree nor disagree | 181 (17.9) | 47 (20.6) |  |
| Agree | 102 (10.1) | 48 (21.1) |  |
| Strongly agree | 82 (8.1) | 72 (31.6) |  |
| ^a^Missing, n | 783 | 3 |  |
| I would rather protect myself by other means (physical distancing, hand hygiene, wearing a mask) than be vaccinated |  |  |  |
| Strongly disagree | 471 (46.5) | 32 (13.9) | <0.001 |
| Disagree | 225 (22.2) | 32 (13.9) |  |
| Neither agree nor disagree | 164 (16.2) | 69 (30.0) |  |
| Agree | 95 (9.4) | 52 (22.6) |  |
| Strongly agree | 57 (5.6) | 45 (19.6) |  |
| ^a^Missing, n | 785 | 1 |  |
| Medical reasons (e.g., allergies) prevent me from being vaccinated |  |  |  |
| Strongly disagree | 900 (88.6) | 163 (71.2) | <0.001 |
| Disagree | 58 (5.7) | 23 (10.0) |  |
| Neither agree nor disagree | 31 (3.1) | 19 (8.3) |  |
| Agree | 14 (1.4) | 8 (3.5) |  |
| Strongly agree | 13 (1.3) | 16 (6.79 |  |
| ^a^Missing, n |  |  |  |
| The coronavirus vaccine has been developed too quickly |  |  |  |
| Strongly disagree | 254 (25.0) | 25 (10.9) | <0.001 |
| Disagree | 225 (22.1) | 22 (9.6) |  |
| Neither agree nor disagree | 268 (26.4) | 46 (20.1) |  |
| Agree | 147 (14.5) | 54 (23.6) |  |
| Strongly agree | 123 (12.1) | 82 (35.8) |  |
| ^a^Missing, n | 780 | 2 |  |
| I feel overwhelmed by information on the coronavirus vaccine |  |  |  |
| Strongly disagree | 316 (31.1) | 46 (20.1) | <0.001 |
| Disagree | 243 (23.9) | 53 (23.1) |  |
| Neither agree nor disagree | 243 (23.9) | 48 (21.0) |  |
| Agree | 134 (13.2) | 37 (16.2) |  |
| Strongly agree | 81 (8.0) | 45 (19.7) |  |
| ^a^Missing, n | 780 | 2 |  |

^a^Participants with missing data were not asked the questions assessing vaccination-related beliefs and attitudes and vaccination acceptance at baseline as they were already vaccinated at baseline.

**Supplementary Table 3. Socio-demographic statistics for the overall study sample and the subsample with full data**

| **Variables administered to everyone at baseline** | Overall study sample that provided data on vaccination  (N= 10,642) | Subsample with full data  (N= 2,028) |
| --- | --- | --- |
| Age, M (SD) | 55.65 (16.46) | 55.05 (15.81) |
| Missing | 400 | 0 |
| Gender, n (%) |  |  |
| Men | 5474 (51.44) | 1013 (49.95) |
| Women | 4767 (44.79) | 1013 (49.95) |
| Other | 7 (0.07) | 2 (0.1) |
| Missing | 394 | 0 |
| Education, n (%) |  |  |
| Primary | 498 (4.88) | 62 (3.06) |
| Secondary | 4749 (46.51) | 983 (48.47) |
| Tertiary | 4964 (48.61) | 983 (48.47) |
| Missing, n | 251 | 0 |
| Number of chronic health conditions, M (SD) | .56 (.87) | .56 (.85) |
| Missing, n | 0 | 0 |
| Work status, n (%) |  |  |
| Employed | 4663 (45.54) | 1017 (50.15) |
| Retired | 3737 (36.50) | 748 (36.88) |
| Not employed | 1839 (17.96) | 263 (12.97) |
| Missing | 403 | 0 |
| Income, n (%) |  |  |
| CHF 0 – 6'000 | 3340 (34.83) | 655 (32.30) |
| CHF > 6'000 – 12’000 | 4479 (46.70) | 1000 (49.31) |
| CHF > 12’000 – 18’000 | 1232 (12.85) | 272 (13.41) |
| CHF > 18’000 | 539 (5.62) | 101 (4.98) |
| Missing, n | 1052 | 0 |
| Language spoken in the study site, (%) |  |  |
| German | 6051 (56.86) | 1237 (61.0) |
| French | 3625 (34.06) | 791 (39.0) |
| Italian | 966 (9.08) | 0 |
